# Bodily maps of emotions and pain: tactile and hedonic sensitivity in healthy controls and patients experiencing chronic pain

**DOI:** 10.1097/j.pain.0000000000003027

**Published:** 2023-09-06

**Authors:** Juhani Ojala, Juulia T. Suvilehto, Lauri Nummenmaa, Eija Kalso

**Affiliations:** a Department of Anaesthesiology, Intensive Care and Pain Medicine, Helsinki University Hospital and University of Helsinki, Helsinki, Finland; b Center for Social and Affective Neuroscience, Department of Biomedical and Clinical Sciences, Linköping University, Sweden; c AI Competence Center, Sahlgrenska University Hospital, Region Västra Götaland, Gothenburg, Sweden; d Turku PET Centre, University of Turku, Finland; e Turku University Hospital and University of Turku, Finland; f Department of Psychology, University of Turku, Finland; g SleepWell Research Programme, Faculty of Medicine, University of Helsinki

**Keywords:** Embodiment, Somatosensation, Interoception, Emotions, Pain assessment, Chronic pain, CRPS, Fibromyalgia, Low back pain, Neuropathic pain, Bodily sensation maps

## Abstract

Pain is an unpleasant sensory and emotional experience. Both pain and emotions are warning signals against outside harm. Interoception, bodily sensations of emotions can be assessed with the emBODY tool where participants colour the body parts where they feel different emotions. Bodily maps of emotions (BMoE) have been shown to be similar between healthy individuals independent of age, sex, cultural background, and language. We used this tool to analyze how these body maps may differ between healthy controls and patients with persistent pain. We recruited 118 patients with chronic pain. An algorithm-selected matched controls from 2348 individuals who were recruited through social media, message boards, and student mailing lists. After providing background information, the participants completed the bodily topography colouring tasks with the emBODY tool using tablets (patients) and online using their own devices (controls), for pain, sensitivity for tactile, nociceptive and hedonic stimuli, and for the 6 basic emotions and a neutral state. Patients with pain coloured significantly larger areas for pain and more negative emotions. On the whole, their BMoEs were dampened compared with healthy controls. They also coloured more areas for nociceptive but not for tactile or hedonic sensitivity. Patients and controls marked different body areas as sensitive to nociceptive and tactile stimulation, but there was no difference in sensitivity to hedonic touch. Our findings suggest that emotional processing changes when pain persists, and this can be assessed with these colouring tasks. BMoEs may offer a new approach to assessing pain.

## Abstract 2

Supplemental Digital Content is Available in the Text.

Bodily maps of emotions, pain, and sensitivities were evaluated in patients with chronic pain and healthy controls. Bodily maps of emotions of patients were dampened and compared with controls.

## 1. Introduction

Emotions prepare us for action through allostatic mechanisms. They manage musculoskeletal, neuroendocrine, and autonomic nervous system activation for promoting survival.^15^ Subjective, phenomenological experience of emotions has been proposed to emerge through somatosensory and interoceptive mechanisms, which track the emotion-dependent changes in the organism's physiological state.^7,21^ These subjective sensations act as warning signals and allow the individual to use higher-order, cognitive strategies for resolving the encountered survival challenges. Pain is an unpleasant sensory and emotional experience associated with, or resembling that associated with, actual or potential tissue damage.^24^ As both pain and emotions are centrally corporal phenomena whose function is to warn the individual about somatic or psychological harm, resolving the links between bodily experience of emotions and pain would be critical for the understanding of the biopsychosocial mechanisms and expression of pain.

Acute pain is a strong alarm signal that first activates the classic fight or flight responses and then prepares the body to protect itself. Pain has a strong aversive affective component, and both chronic and acute pain profoundly influence emotional functioning. For example, greater pain associates with increased emotional stress, limited emotional awareness, and emotion expression.^19^ Follow-up fMRI studies have shown that the processing of information associated with painful episodes shifts from nociceptive to emotional brain circuits.^12^

Pain and bodily sensations of emotions stemming mainly from visceral and autonomic inputs can be quantified using bodily sensation maps (BSMs). In this technique, the subjects are asked to indicate on a human figure the regions where they experience alterations in bodily functioning during specific imagined or induced emotional states.^21,22,30^ The resultant BSMs likely reflect a compound measure of the effects of autonomic nervous system, skeletomuscular, and visceral sensations, which the individuals cannot separate. Thus, although the specific physiological systems underlying these sensations cannot be directly determined, the net sensations arising from multiple physiological systems during different emotions are topographically distinct.^21^ Bodily sensation maps are concordant across sexes and consistent across lifespan, with only gradual weakening during ageing.^32^ They may also be diagnostically informative because BSMs differ between, for example, healthy controls and patients with schizophrenia.^31^

The BSMs provide an interesting parallel with pain drawings or sensory maps that are commonly used in the clinical evaluation of pain.^2,28,29^ Considering the central role of emotions in pain, contrasting the bodily experience and specific emotions by comparing emotional body maps with the sensory maps could provide new insights into the interplay between somatosensation, emotion, and pain. In this study, we investigated the bodily sensations of pain and mapped subjective imagined topographical sensitivity to nociceptive, tactile, and hedonic somatosensation and bodily experience of emotions in patients with chronic pain and healthy controls. Our hypotheses were that chronic pain associates with enhanced sensitivity to tactile stimulation but reduced sensitivity to hedonic touch and that the patients might have different sensory and emotional body maps reflecting their pain condition. This information could provide new insight into the interaction of pain and interoception, and how this knowledge could be used to better understand patients with persistent pain.

## 2. Methods

### 2.1. Participants

The participants consisted of a group of patients with chronic pain and a control group representing the general population. The study was approved by the Ethics Committee of the Helsinki and Uusimaa Hospital District (HUS/3382/2017).

#### 2.1.1. Patients with chronic pain

**Figure 1.**
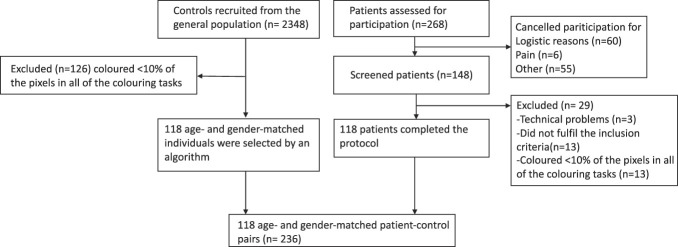
Flowchart showing the participant acquisition for the study, shown separately for patients with chronic pain and the healthy controls.

The patients were recruited from the Multidisciplinary Pain Clinic of the Helsinki University Hospital. Inclusion criteria were older than 18 years of age, fluency in Finnish, and ability to use the emBODY tool^21^ on an iPad. The patients recruited for this study had a pain diagnosis (complex regional pain syndrome [CRPS] type I or II, fibromyalgia, neuropathic pain, or low back pain) confirmed by an experienced pain physician. Patients who had major psychiatric conditions, active cancer, or who used strong opioids were excluded. All eligible patients were invited to participate. However, as the patients were recruited by a part-time research nurse, recruitment was possible only on days when she was at work (roughly one day a week). All participants provided a written informed consent. The final sample (Fig. 1) consisted of 118 patients.

#### 2.1.2. The control group

A large sample of participants (n = 2348) from the general population was recruited using social media, message boards, and student mailing lists. The participants from the general population completed the study entirely online using their own devices (tablet or computer). Only individuals ≥18 years of age were recruited, and the study was only available in Finnish. There were no other exclusion criteria.

From this sample, an algorithm selected age-matched and gender-matched controls for patients with pain (n = 118) so that the age differential between the participant and control was the smallest possible while prioritizing controls with low current pain. Only controls who answered “no” to the question whether they experienced a chronic pain condition were considered for the matching. Moreover, only controls who reported acute pain intensity less than 3 on a numerical pain scale (NRS 0-10) and BPI (now and 24 hours mean) below 5 were included (Fig. 1).

As requested by one of the reviewers, we reanalyzed the data also with a healthy control group who endorsed only a 0, 1, or 2 on the BPI average and now items. This reduced the number of controls to 106 in the additional analyses.

### 2.2. Procedure

First, all participants provided demographic information about age, gender, and education level. They were then asked whether they had any current acute pain within the past 24 hours. In case of a positive answer, they were asked to fill the Brief Pain Inventory (BPI).^5^ The short form of BPI evaluates pain intensity during the previous 24 hours and its impact on mood, walking, working, relationships, sleep, joy of life, and life in general.

All participants were also asked whether they had a history of repeated and/or very painful migraine, headache, abdominal pain, back or neck pain, limb or joint pain, or menstrual pain and whether they had or had had any pain that had lasted for more than 3 months. The participants were asked to rate their current pain on a scale from 0 to 10 (0 indicating no pain and 10 the worst possible pain). They were also asked to report the frequency of use of over-the-counter and prescription analgesics and other medications having an effect on the central nervous system such as antidepressants or anxiolytics.

The participants also evaluated their current emotional state by rating how much, at the moment of filling in the survey, they felt anger, fear, disgust, happiness, sadness, surprise, anxiety, and depression. These ratings were done on a scale ranging from 0 (none) to 10 (as much as possible).

After providing background information, the participants completed the bodily topography colouring task with the emBODY tool^21^ with iPad pro tablets (patients) and online using their own devices (controls). The participants were first given instructions on how to use the colouring tool and then shown a pair of 2-dimensional human silhouettes with a prompt explaining what they should colour in the image (see below). The participant was able to freely colour anywhere on the web page similarly as one does in a colouring book. When the participant was happy with the colouring, he/she clicked on a button marked “ready” and was shown a new pair of blank silhouettes and a new prompt.

There were 3 categories of colouring tasks, each with multiple coloring tasks, ie, bodies to be coloured in. The order of the categories was always the same, but the order of individual colouring tasks inside each category was randomized between the participants.

The tasks were as follows. (1) emotional experience in the body: The participants were asked to colour where in their body they typically experience increasing or decreasing activation while having these emotions. One silhouette was used for activations and another for decreasing activations. No reference to any internal or external stimulus was used. This was done on 7 pairs of figures accompanied by 6 emotional states (anger, fear, disgust, happiness, sadness, surprise) and a neutral state. No distinction was made between the front and the back of the body on the emotion-colouring tasks because emotions stem from deep visceral and autonomic activity, which does not clearly locate on surface (front/back) of the body. The silhouettes did not include any clues as to which side of the body they represented. (2) Pain maps: The participants were shown silhouettes depicting the front (left side of the screen) and back (right side of the screen) of the body. They were asked to colour in 2 pairs of images: one for areas where they currently felt pain and one for areas where they felt long-lasting and/or frequent pain. (3) Sensitivity maps: The participants were shown 3 pairs of images with front and back of the body, as above. The tasks were to colour the areas that they typically experienced as particularly sensitive to pain (“nociceptive sensitivity”), where the participants could easily feel even a light touch (“tactile sensitivity”), and areas whose touching feels pleasant to the participant (“hedonic sensitivity”). The participants were not asked to self-stimulate these areas.

Patients with pain, but not the controls, had an option to indicate that they had left an image empty on purpose. This was to ensure that we were able to differentiate accidently empty bodies (arising, eg, from clicking “ready” twice in a row without meaning to) from intentionally empty bodies. Because the general population sample did not have this option, we treated all empty bodies by the controls as intentionally empty to minimise the potential difference this feature might cause. For patients with pain, body maps that had not been intentionally marked empty and did not contain any coloured pixels were treated as missing data and dropped from analyses.

The patients coloured the maps and answered the questionnaires in a quiet area while waiting for their appointment at the Pain Clinic. None of the participant had just had or was going to have an interventional procedure.

### 2.3. Data processing

The data were collected using an online system adapted from the original emBODY tool (https://version.aalto.fi/gitlab/eglerean/embody). Although the participants were able to repeatedly colour over a single location, increasing the opacity of the colouring, we converted the colouring to binary maps (yes/no) for the analyses to minimize potential confounds stemming from using different devices/device types (mouse, touchpad, touch screen) on the coloring. The emotion maps, which reported activation/deactivation, were combined such that the deactivation and activation maps were overlayed, with deactivations marked with −1, activations with 1, and uncoloured pixels marked with 0. We also defined 8 regions of interest (ROIs) in the body. These were head, shoulders, arms, hands, upper torso, lower torso, legs, and feet (see Suppl Fig. 2 for a visual depiction, available at http://links.lww.com/PAIN/B903).

#### 2.3.1. Quality control

To ensure the quality of the colouring data, the colouring maps of all participants were first screened visually to exclude any that were clearly outside the body outline. No participants were excluded for this reason. To minimize the effect of problems with using the colouring tool, we excluded all participants who had not coloured in at least 10% of the pixels in at least one of the colouring tasks (any emotion, any sensitivity, or any pain map). This led to the exclusion of 126 controls and 13 patients with pain. Quality control was performed before matching the controls with the patients. Thus, all the matched controls were included in the data set.

#### 2.3.2. Statistical analyses

Statistical analyses were conducted using *Python* 3.6.9 for image-based analyses and R 3.6.3 for other analyses. The maps derived from the colouring tool were visualized as proportion of coloured pixels (ie, how large a proportion of participants coloured that specific pixel). Statistical comparisons for the maps were done using mass univariate 2-sample *t* test (in emotion tasks) or two-proportion z-test (in pain and sensitivity tasks). The resulting comparison maps were corrected for false discovery rate (FDR)—corrected for the number of pixels within the silhouette outline (45,443 pixels for the emotion maps, 90,931 pixels for the 2-sided maps). Only significantly different pixels (after FDR correction) are shown in visualizations. False discovery rate correction was used for the comparisons of the body maps because it is somewhat less stringent than the alternatives and the number of pixels is very high. A more conservative multiple comparison correction (Holm–Bonferroni) was used for other statistical analyses.

The number of coloured pixels for each participant and each map was computed, and it is presented as proportion of the whole body. Thus, if the participant had coloured in the entire silhouette (in emotion tasks) or both of the silhouettes (in pain and sensitivity tasks), the coloured proportion would be 1, whereas it would be 0 if the body was left blank. We counted the proportion of the coloured area for the anatomically defined regions of interest similarly.

The number of coloured pixels across emotions in the control and patient groups was compared with 2-way between-subject and within-subject ANOVA on trimmed means, using the bwtrim function from WRS2 R-package because of non-normal data. Optimally, we would have used a three-way between-subject and within-subject subjects ANOVA to test for region-of-interest–specific analyses, but as the bwtrim function is only defined for two-way ANOVA, we instead ran the analyses for each ROI specifically.

## 3. Results

One hundred eighteen patients (42 CRPS, 24 neuropathic, 12 fibromyalgia, 32 low back, and 8 other/combination) and 118 age-matched and gender-matched controls from the total sample of 2348 controls were included (Fig. 1). None of the controls reported having experienced long-lasting pain (>3 months duration) or experiencing significant pain at the moment (Suppl. Table 1, available at http://links.lww.com/PAIN/B903). Most controls (n = 98) reported experiencing no pain at the time of filling in the survey, 10 reported pain intensity of 1 (on a scale of 0-10), 6 reported pain intensity of 2, and 4 intensity of 3.

The mean age of the patients was 44.3 years and that of the controls was 43.9 years (NS). In both groups, 87% were women. The participants in the control group had higher education level compared with the patients (Suppl. Table 2, available at http://links.lww.com/PAIN/B903). The use of analgesics is shown in Suppl. table 3 (available at http://links.lww.com/PAIN/B903).

Only a few data points were missing in the responses from the online data collection system. Five pain patients had not coloured the body map for chronic pain and did not confirm that the bodies had been left empty on purpose. These responses were treated as missing. By contrast, data collected from the patients with physical pen-and-paper forms had high levels of missing responses. Only 55 and 58 patients with pain had completed the BDI and STAI questionnaires, respectively, resulting in response rates below 50%. Because of the high amount of missing data, these forms were not analyzed further.

The results of the additional analyses requested by one of the reviewers are shown in Supplemental Additional Analyses, available at http://links.lww.com/PAIN/B903.

### 3.1. Bodily maps for pain

**Figure 2.**
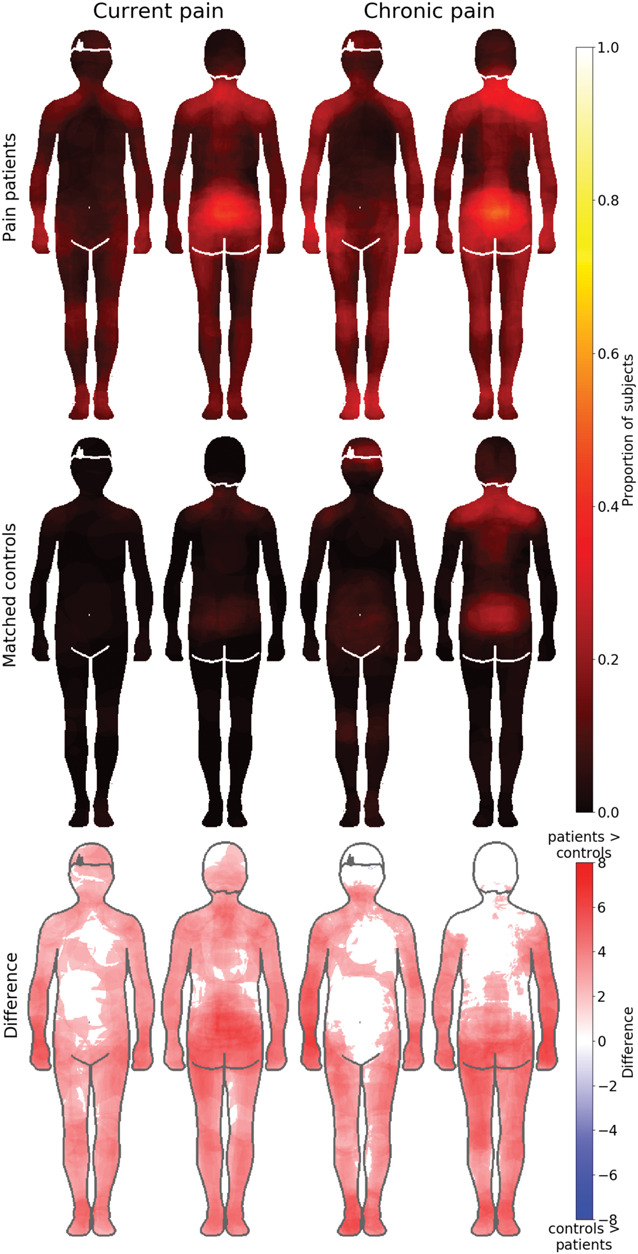
Bodily maps for current (left) and chronic pain (right) across the groups. The chronic pain patient group (top row, N = 113 for chronic pain, N = 118 for current pain) reported significantly more pain in the body than in the control group (middle row N = 118). The figure shows the self-reported pain maps for current pain (2 left-most columns) and chronic pain (2 right-most columns) with patients on the top row and matched controls on the second row. The intensity of the colour represents the proportion of respondents who have coloured that specific pixel. The bottom row shows regions where patients experienced stronger pain than controls. The data on the bottom row are thresholded at *P* < 0.05, FDR corrected.

Figure 2 shows the pain maps for the patient and control groups and the statistical comparison map between the groups. Patients with pain coloured a larger area of the body for both current pain (Mdn = 0.10 of the total body) and chronic pain (Mdn = 0.14 of the total body) conditions than the controls (Mdn = 0 and Mdn = 0.03, respectively). A Mann–Whitney test indicated that this difference was statistically significant for both current (r = 0.762, *P* < 0.001) and chronic (r = 0.588, *P* < 0.001, *P* values Holm–Bonferroni corrected) pain. The effect was also robust on pixel level throughout most of the body (Fig. 2).

**Figure 3.**
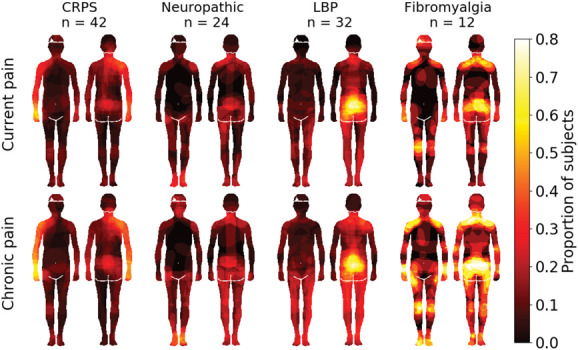
Pain maps across the different patient groups. Pixel intensities show the proportion of individuals, in each group, who had coloured each pixel. N.B.: The maps acquired from patients with CRPS were rotated such that the affected side is always depicted on the right hand side of the drawing. The data are thresholded at *P* < 0.05, FDR corrected.

Maps showing the locations of pain in the subgroups of patients with pain are shown in Figure 3. Both current and chronic pain areas were localised in the arms in the CRPS patients, lower back area in patients with low back pain (LBP), in the feet in the NP patients, and across multiple locations in the FM patients. Patients with NP marked pain to the neck area and to the lower back but most to the feet.

### 3.2. Sensitivity maps

**Figure 4.**
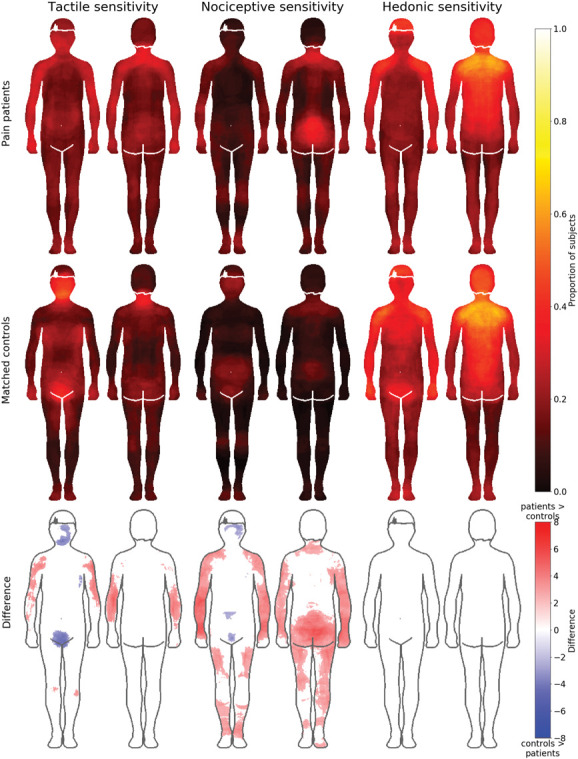
Bodily maps for tactile, nociceptive, and hedonic sensitivity in patients (top row) and controls (middle row). Hot colours indicate higher sensitivity. Bottom row shows the regional differences between the groups. Blue colour indicates higher sensitivity in the control group and red higher sensitivity in the patient group. The data on the bottom row are thresholded at *P* < 0.05, FDR corrected.

Maps for tactile, nociceptive, and hedonic sensitivity in each group are shown in Figure 4. Statistical testing between the groups revealed that in general, patients tended to more consistently colour distal body areas (limbs), whereas the controls more consistently coloured central body areas. For tactile sensitivity, the patients experienced higher sensitivity in limbs, particularly the arms, whereas controls reported higher sensitivity in the face and groin. Patients reported higher nociceptive sensitivity in the limbs and on the back of the torso, whereas healthy controls reported higher sensitivity in the face, lower abdomen, and groin. For hedonic sensitivity, no between-group differences survived FDR correction.

**Figure 5.**
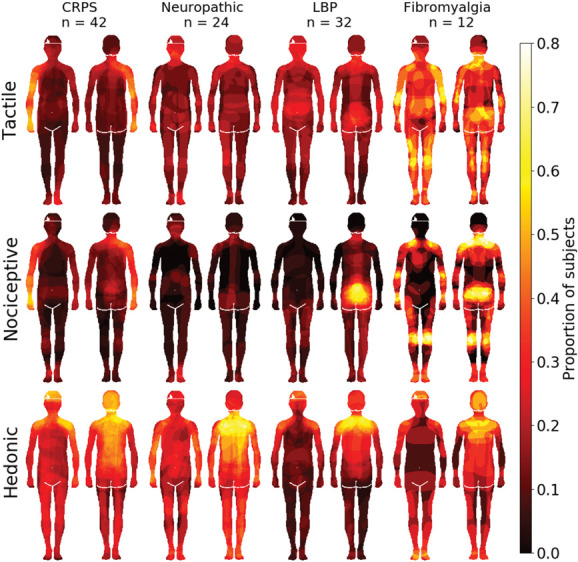
Sensitivity maps across chronic pain subgroups. Pixel intensity shows that the proportion of individuals in each group had coloured each pixel. Note: The maps acquired from patients with CRPS were rotated such that the affected side is always depicted on the right hand side of the drawing.

Figure 5 shows the sensitivity maps separately for the different chronic pain subgroups. Both nociceptive and tactile sensitivities were most intense in the upper arms of patients with CRPS, lower back in patients with low back pain, the feet in patients with NP, and at multiple locations in patients with fibromyalgia.

#### 3.2.1. Region-of-interest analysis for the sensitivity maps

In a complementary region-of-interest (ROI)–based approach, the proportion of coloured pixels across the 8 ROIs (Suppl. Fig. 1, available at http://links.lww.com/PAIN/B903) was compared across the groups. Overall, patients with pain coloured more areas as sensitive for nociception (M = 0.13, SD = 0.12) than the controls (M = 0.06, SD = 0.07; U = 4357.5, *P* < 0.001, r = −0.32). There was no significant difference in the whole-body colouring of tactile sensitivity (U = 6871, *P* = 0.95, r = −0.004) or hedonic sensitivity (U = 7914.5, *P* = 0.056, r = 0.14, *P* values Holm–Bonferroni corrected for multiple comparisons). In two-way between-subject and within-subject ANOVAs on trimmed means for each separate ROI, there were no significant main effects of group (patient or control, *P*s ≥ 0.13). The main effect of condition (tactile, hedonic or nociceptive sensitivity) was significant for all ROIs (*P*s ≤ 0.02) apart from legs (*P* = 0.41) and feet (*P* = 0.25). The only significant interaction was for the lower torso (*P* = 0.007). In pair-wise Mann–Whitney U tests between the groups, for each ROI and condition, the only significant differences were found in the reporting of nociceptive sensitivity in shoulders (U= 9030.5, *P* = 0.00,005), arms (U = 9373.0, *P* = 0.0,000,003), hands (U = 8932.5, *P* = 0.00,001), and legs (U = 8440.0, *P* = 0.006). In all of these cases, patients had coloured in more areas as being sensitive to nociception than controls.

As visual inspection of the hedonic sensitivity maps seemed to suggest that patients with LBP and FM may have less hedonic sensitivity compared with the other pain patients, we performed a secondary analysis. The group difference in hedonic sensitivity between patients with LBP and FM, and those with other pain was tested with Welch two-sample *t* test. Patients with LBP and FM (mean = 0.2148) did not significantly differ from patients with other pain (mean = 0.2790), t(72.761) = −1.2042, *P* = 0.2324.

### 3.3. Bodily maps for the basic emotions

**Figure 6.**
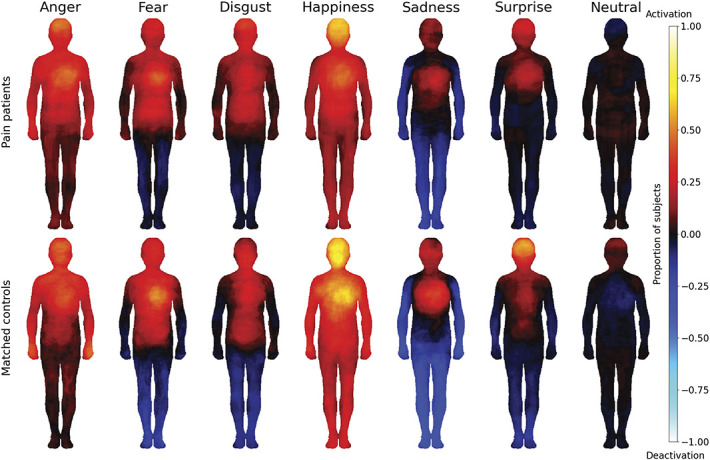
Bodily maps for emotions of patients with chronic pain (top row) and matched controls (bottom row). Hot colours represent experienced activation, and blue colours deactivation.

Bodily maps of the basic emotions and the neutral state for both groups are shown in Figure 6. The proportion of coloured body area was largest for happiness (mean 0.316, SD 0.265) and sadness (mean 0.305, SD 0.256). No regions survived multiple comparison correction when the emotion-specific maps were compared across the groups.

**Figure 7.**
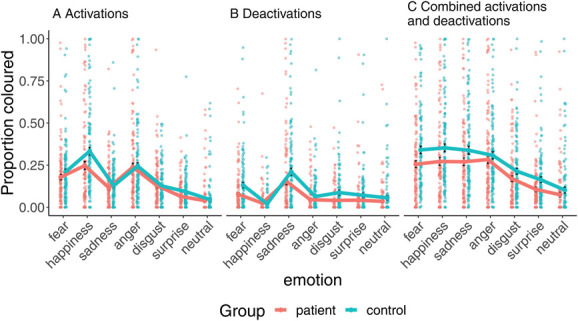
The proportion of the body area where emotion-specific activations (A), deactivations (B), or either activations or deactivations (C) were felt. Whiskers show standard error of the mean.

In a complementary analysis, the total coloured bodily area was compared between patients with pain and controls (Fig. 7). A 2-way between-subject and within-subject ANOVA on trimmed means was conducted with a between-subject factor as group membership (2 levels: patient and control) and within-subject factor as emotion (7 levels). There was a significant main effect for group (F(1375.64) = 30.94 and *P* < 0.0001), such that on average, the bodily area coloured by patients with pain (mean proportion coloured = 0.2, sd = 0.23) was smaller than the area coloured by controls (mean proportion coloured = 0.26, sd = 0.24). There was also a significant main effect for emotion (F(6214.94) = 72.40 and *P* < 0.0001). There was no significant interaction between group and emotion (F(6214.00) = 1.49 and *P* = 0.1831). The least coloured body area was in the neutral emotional state (mean 0.092, SD 0.179), followed by surprise (mean 0.135, SD 0.174) and disgust (mean 0.193, SD 0.191).

Similar main effects of group and emotion were also found when analyzing activations (F(1, 261.07) = 14.18, *P* = 0.0006; F(6193.64) = 157.59, *P* < 0.0001) or deactivations (F(1375.64) = 30.93, *P* < 0.0001; F(6214.95) = 72.40, *P* < 0.0001) separately. The interaction between group and emotion was significant for deactivations (F(6191.78) = 5.82, *P* = 0.0001) but not for activations (F(6193.90) = 2.27, *P* = 0.077, all *P* values Holm–Bonferroni corrected).

#### 3.3.1. Region-of-interest analysis for the bodily maps of emotions

To test whether the aggregate effect was localized on a specific body area, we analyzed the total number of coloured (activation or deactivation) pixels in 8 anatomically defined regions of interest (ROI). In the ROI analysis (Suppl. Fig. 2, available at http://links.lww.com/PAIN/B903), we ran a 2-way between-subject and within-subject ANOVA on trimmed means for each body part, separately. There was a significant main effect of group for each ROI (all *P*s < 0.005, Holm–Bonferroni corrected) with stronger bodily feelings in the controls vs patients. For all ROIs, there was also a significant main effect of emotion (all *P*s < 0.001, Holm–Bonferroni corrected). There were no significant interactions. When we reanalyzed the data with the more clearly pain-free controls (n = 106), 3 body areas showed a weak but statistically significant interaction between emotion and group membership (Suppl. Additional Analyses, available at http://links.lww.com/PAIN/B903).

### 3.4. Self-reported emotional experience

Finally, we analyzed the self-reported experience of basic emotions, depression, anxiety, and pain. In a Two-way between-subject and within-subject ANOVA on trimmed means, there was a significant main effect of group F(1, 84.26) = 96.60, *P* < 0.001, such that on average, patients reported higher intensities (mean 3.262, SD 2.85) than controls (mean 1.389, SD 2.327) (Suppl. Fig. 3, available at http://links.lww.com/PAIN/B903). There was also a significant main effect of emotion type F(8, 73.39) = 46.19, *P*< 0.001 and a significant interaction F(8, 73.39) = 38.89, *P* < 0.001. All post hoc pairwise comparisons between the groups indicated significant differences between the groups' responses. In all of the negative emotional states, as well as surprise and pain, the patients reported experiencing more of that emotion than the controls (*Ps* < 7 × 10^−07^). Conversely, controls reported experiencing more happiness (M = 5.3, SD = 2.5) than patients (M = 4.5, SD = 2.3) (U = 5488.0, *P* = 0.0046, Holm–Bonferroni corrected for multiple comparisons).

Associations between pain, tactile, nociceptive, and hedonic sensitivity, and current emotional state in both groups are shown in Suppl. Figure 4 (available at http://links.lww.com/PAIN/B903). Most correlations were similar between the 2 groups. Clear differences between groups can be seen in the correlation between fear and the extent of current pain. In the patient group, this correlation is not significant (r = −0.013, *P* = 1), whereas in the control group, there is a significant moderate correlation between the two (r = 0.42, *P* = 0.0002). This difference is statistically significant (Fisher z = −3.53, *P* = 0.043). Moreover, the correlation between the extent of current and chronic pain is significant in both groups, but it is significantly stronger in patients with pain (r = 0.74, *P* = 6 × 10^^−19^) than in controls (r = 0.4, *P* = 0.0006) (Fisher z = 3.98, *P* = 0.007, all *P* values Holm–Bonferroni adjusted for multiple comparisons).

### 3.5. Correlations between brief pain inventory, positive emotions, and sensations (secondary analysis)

We analyzed a possible association between pain and positive emotions and sensations in patients with pain. For the analysis, we condensed the BPI interference items to a “mean interference” value. After multiple comparison correction with Holm–Bonferroni, there was a significant negative correlation between BPI mean interference and feeling of happiness at the moment (r = −0.42, *P*(adjusted)= 0.000075). The area of the body coloured in the happiness emotion and hedonic sensitivity tasks did not significantly correlate with any of the BPI responses.

## 4. Discussion

### 4.1. Main results

Our main finding was that bodily experience of emotion and pain as well as self-reported nociceptive, tactile, and hedonic sensitivity differ across chronic pain and matched healthy controls. Patients with pain showed higher sensitivity for pain and tactile sensitivity in their respective pain areas, with similar hedonic sensitivity compared with the controls. The bodily representations of emotions were dampened in the patients, although they reported significantly stronger negative emotions.

As expected, the patients felt both current and chronic pain in larger body areas than controls. More interestingly, the patients reported higher tactile and nociceptive sensitivity in the limbs and higher nociceptive sensitivity in the low back in comparison with the controls. Conversely, the controls reported higher sensitivity in facial and pelvic areas. This altered bodily sensitivity also extended in the emotional domain but in the opposite direction. Patients with pain reported generally reduced embodied experience of emotions in comparison with the controls. Overall, these data highlight the interaction between the emotion and pain circuits and reveal how chronic pain may lead to perturbations in the somatovisceral experience of emotions in the body for the first time.

### 4.2. Pain and nociceptive sensitivity

As expected, patients with pain reported more pain than controls in the questionnaires and in the colouring task. The most common areas where pain was felt in both groups were lower back and the shoulder–neck regions. These areas represent the most common sites of both acute and chronic musculoskeletal pain.^34^ The pain drawings also show that patients with CRPS felt more pain in the upper arms, those with low back pain in the low back area, whereas those with fibromyalgia experienced more pain in both all limbs, shoulders, and particularly lower back. These results agree with the clinical picture of each condition because the CRPS was always in the upper arm of these patients and patients with fibromyalgia often also have low back pain.^4^ In the body maps for nociceptive sensitivity, patients with low back pain painted strong sensitivity in the low back area, those with CPRS in the upper arms, and those with fibromyalgia over the whole body. The NP patients indicated the feet as the most pain sensitive body part, suggesting polyneuropathy as a possible cause. These results agree with the clinical picture of these pain conditions, which have been shown to associate with reduced thresholds for particularly mechanical nociceptive sensitivity indicative of central sensitization.^25,35^

### 4.3. Tactile and hedonic sensitivity

Our hypothesis was that patients with chronic pain would have enhanced sensitivity not only to nociceptive but also to tactile sensitivity and decreased sensitivity to hedonic touch. Contrary to our hypothesis, the amount of coloured pixels in both tactile and hedonic body maps was similar between the groups. However, the patients and controls had different topographical maps for these sensitivities. Patients reported higher tactile sensitivity in the arms and controls in the face and groin. The density of tactile receptors is highest in the face and palms, which might explain the results of the control group.^6^ Of the different chronic pain patient groups, patients with CPRS reported highest sensitivity in the arms and patients with fibromyalgia across multiple bodily areas, agreeing with the clinical picture of hyperesthesia being an important component of these conditions.^11,27^

Contrary to our hypothesis, sensitivity to hedonic touch was similar in both controls and patients with pain.

This would suggest that hedonic touch was preserved in patients with persistent pain providing, further evidence for the important role of affective touch. Hedonic or affective touch is mediated by a class of low-threshold mechanosensitive afferents, the C-tactile afferents (CTs).^20^Hedonic touch is rewarding because stroking of the skin produces a pleasant emotion.^8,18^ Importantly, pleasant skin-to-skin contact promotes interpersonal touch and affiliative behaviour. Affective touch may also contribute to interoception, the sense of our embodied psychological “self.”^17^

A previous study assessing brain basis of gentle touch perception using fMRI in patients with fibromyalgia^3^ suggested that patients with fibromyalgia exhibited anhedonia to gentle touch with intact early-stage sensory processing but dysfunctional evaluative processing. In this study, however, also patients with fibromyalgia indicated high hedonic sensitivity across the whole body. Similar to other patients with pain and controls, they indicated highest sensitivity in the shoulder and upper back areas.

Preference for touch in these sites is in line with earlier research. Social touch on the back is rated as more pleasant than on other locations on the body.^33^ These areas are also most closely related to variation of social touch in different social relationships,^30^ which suggests that they are important for social interactions. Although the exact distribution of CT receptors in the human skin is not known, C-fibre low-threshold mechanoreceptors, which are the rodent equivalent of C-tactile afferents, have been found to have a denser distribution in areas corresponding to the back and shoulders.^16^

### 4.4. Emotional body maps and pain

Both controls and patients indicated experiencing emotions in comparable bodily regions, and these topographies accord well with those found in prior studies.^21,22^ However, statistical analysis revealed that the magnitude of embodied emotional experience was significantly lower in the patient group. This effect was consistent across emotions and also across most bodily regions. Although the bodily experience of emotions was significantly dampened in the patients, they reported substantially higher levels of negative emotional experiences (anxiety, depression, fear, anger, etc) than controls. It is possible that the constant nociceptive signaling in chronic pain interferes with afferent visceral signals conveyed by the spinal and cranial nerves that drive the generation of subjective emotional states.^1^ Somatosensory and visceral inputs are not the only determinants of emotional states, and individual with eg, pure autonomic failure have phenomenological experience of emotions.^13^ The current data could thus indicate that peripheral sensory exteroceptive information because of persistent pain may overshadow interoceptive accuracy, shifting the emotional experience from the periphery towards central domains.^14,23^This shift could be because of automatic downregulation of the somatosensory and nociceptive inputs to alleviate the experience of pain.

Our results are supported to some extent by a previous study that also used a manikin to assess pleasant and unpleasant sensations in patients with chronic pain and healthy controls.^10^ The study showed that patients with chronic pain indicated significantly more unpleasant emotions in the manikin compared with healthy controls. However, because of the differences in methodology, we cannot directly compare the results. The study by Hanley and Garland assessed only unpleasant and pleasant sensations in patients with chronic pain who were all on opioid therapy. Our study subjects reported 6 basic emotions and one neutral sensation. None of our patients were on opioids, which are known to have significant effects on mood. For example, long-term opioid treatment significantly increases the risk for major depression disorder and anxiety and stress-related disorders.^26^

### 4.5. Strengths and limitations

The main strength of the study is the careful multimethod evaluation of pain and topographical evaluation of the experience of both pain and emotions. The bodily emotion mapping tool was also established as a potential novel marker for affect dysregulation in chronic pain conditions.

No sensory manipulation or intervention was used in our trial. This could be seen as a limitation. However, this was not the focus of this study, which aimed to assess the subjective sensation without external sensory manipulations. However, this is an interesting topic for future research. It should be noted that the stimulations and who performs them (the person her/himself or another person) need careful consideration. Previous reports suggest that individuals differ in their preferences regarding who can touch them and where.^30^

The patient groups with different types of pain were too small reducing the statistical power of the comparisons. Thus, future studies with larger groups of different types of pain are needed. Missing data from psychological questionnaires did not allow assessing the psychological questionnaires in the patients. However, they did provide sufficient information about mood using the emBODY tool.

### 4.6. Future

Evaluation of pain, its intensity and interference, still relies mainly on simple questionnaires and scales. Emotional states and bodily sensations of emotions are emerging new areas of research in pain, both in understanding the mechanisms of pain chronification and as targets for therapy. Digitalized assessment of bodily representations of pain, sensitivity to sensory stimuli, and emotions may offer a new method, which may increase our understanding of different chronic pain conditions, the role interoception, and basic emotions and enhance patient compliance in providing this essential information. This tool may also enable assessment of multidimensional treatment effects in follow-ups. For example, the study by Hanley and Garland showed, using a sensation manikin, that an intervention (Mindfulness-Oriented Recovery Enhancement) significantly increased the pleasant sensations of patients with chronic pain being treated with opioids^10^. Another previous study suggests that emotional body maps and application of machine learning could also be used to predict future pain.^9^

## Conflict of interest statement

The authors have no conflict of interest to declare.

## Appendix A. Supplemental digital content

Supplemental digital content associated with this article can be found online at http://links.lww.com/PAIN/B903.
