# Supplementary material for: Bodily maps of emotions and pain: tactile and hedonic sensitivity in healthy controls and patients experiencing chronic pain
Source: Pain. 2023 Sep 6;164(12):2665–74. doi: 10.1097/j.pain.0000000000003027 (PMC10652713; doi:10.1097/j.pain.0000000000003027)
Supplement: Supplementary file 1 [file jop-164-2665-s001.pdf]

BMoE 300723

Supplement figures and tables

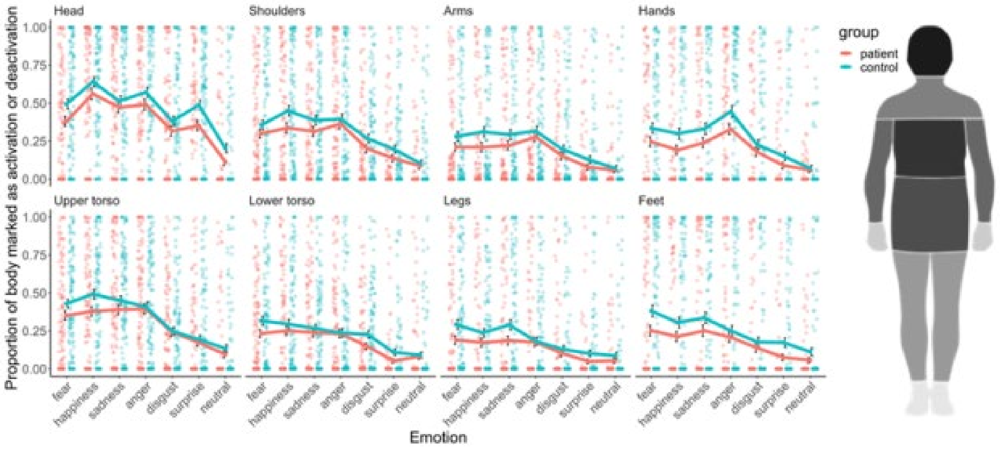

Suppl. Figure 1. Region-of interest analysis for the bodily maps of emotions. The aggregate effect of colouring was repeated for all eight regions of interest. The body was divided into eight separate ROIs and the proportion of pixels coloured within each ROI was counted. The plot depicts for each ROI (panels) the proportion of the ROI coloured (y-axis) for each emotion (x-axis). The points indicate individual participant's responses while the line shows the average for each group. In both, red indicates patients and green controls. Black whiskers show standard error of the mean for that emotion.

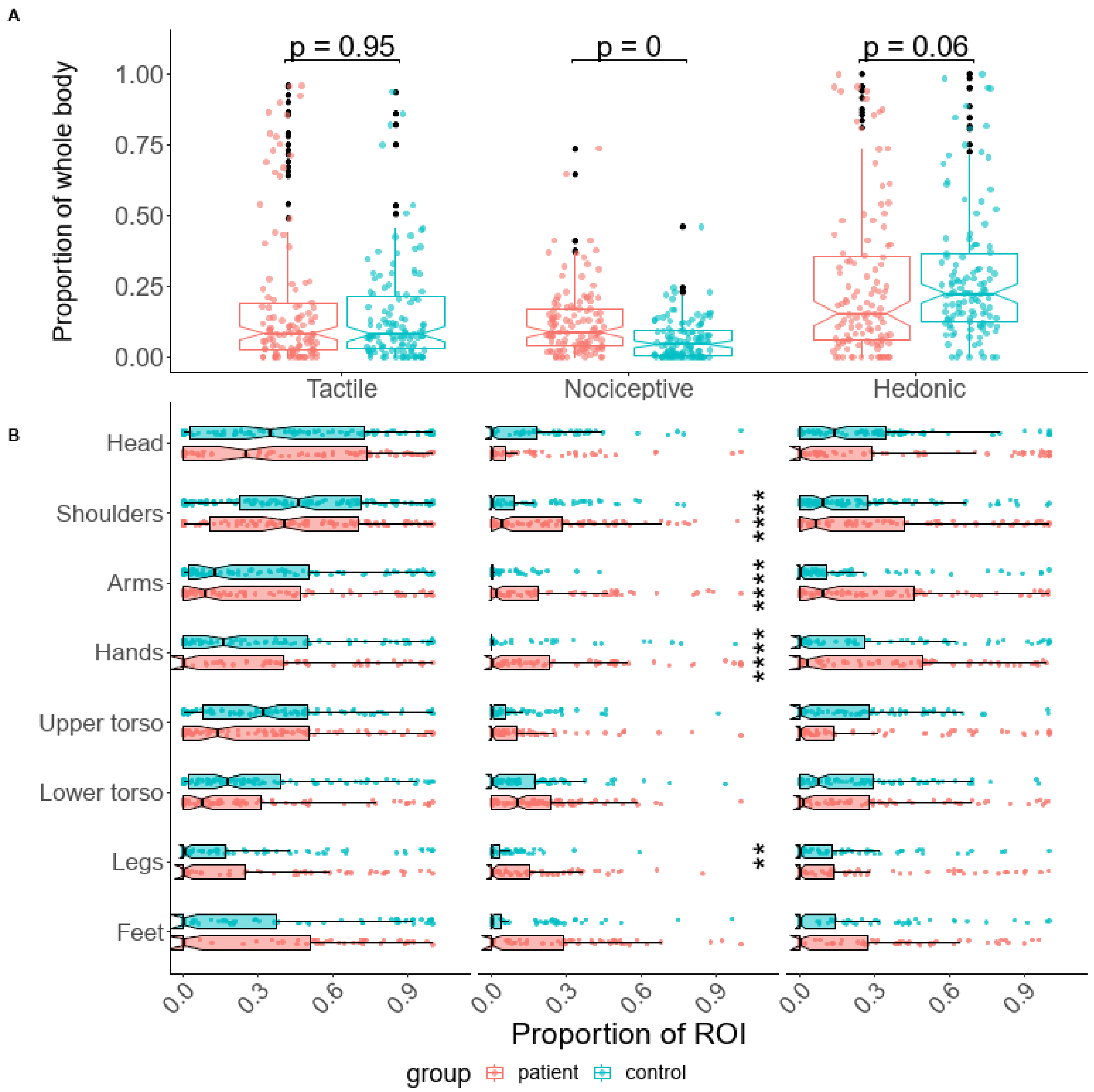

Suppl. Figure 2. Mean total (A) and regional (B) sensitivities for patients and controls. Asterisks indicate statistically significant post-hoc tests dor each region (** p < 0.01, *** p < 0.001, **** p < 0.0001.)

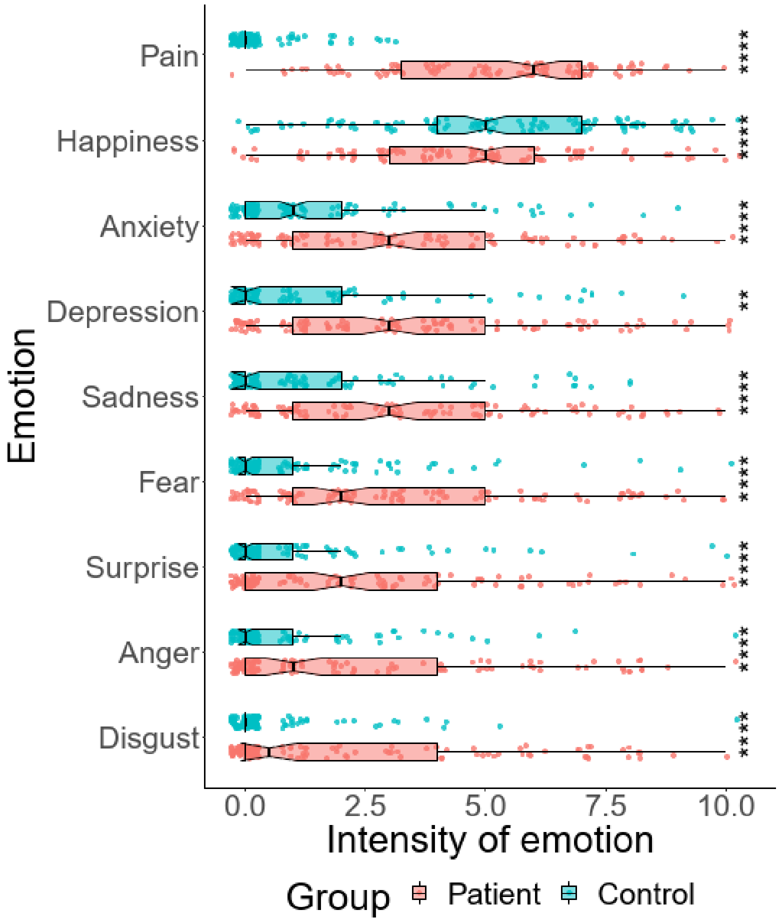

Suppl. Figure 3. Mean intensity of 6 emotions and anxiety, depression, and pain across the groups. Asterisks depict statistical significance level after multiple comparison correction, ** $p < 0.01$, *** $p < 0.001$, **** $p < 0.0001$.

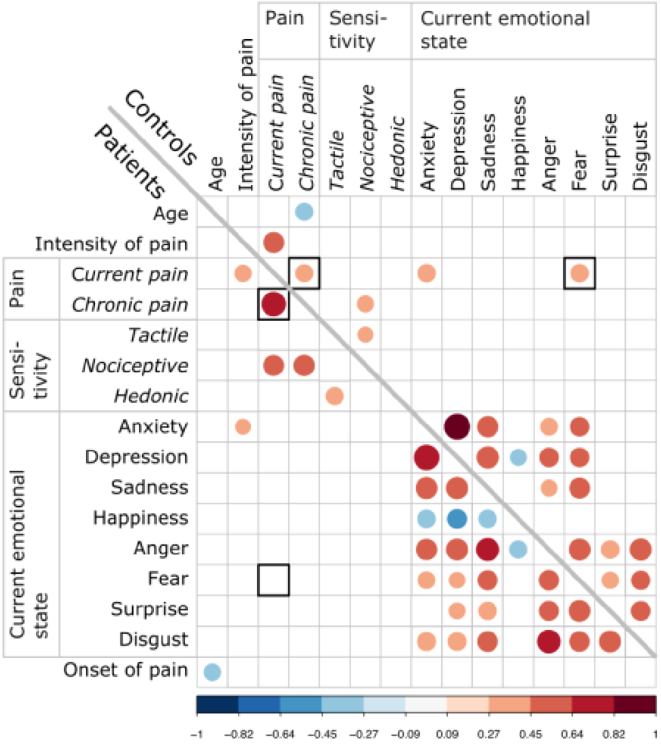

Suppl. Figure 4. Correlations between subjective emotional state and the amount of body area coloured for sensitivity and pain body maps. The correlations for controls are shown on the upper triangular matrix and the patients on the lower triangular matrix. Only significant correlations (p < 0.05 after multiple comparison correction) are shown. Correlations that are significantly different between the two groups are marked with bolded squares.

Pain now	Controls	Patients
No	118 (100%)	2 (1,7%)
Yes	0	116 (98,3%)
**Pain last day**		
No	65 (55%)	0
Yes	53 (45%)	118 (100%)
**Chronic pain**		
No	118 (100%)	2 (1.7%)
Yes	0	116 (98.3%)
**History with Migraine**		
No	75 (64%)	71 (60%)
Yes	22 (19%)	23 (19,5%)
Not within 6 months	21 (17%)	24 (20,5%)
**History with Headache**		
No	83 (70%)	71 (60%)
Yes	22 (19%)	36 (31%)
Not within 6 months	13 (11%)	11 (9%)
**History with Abdominal Pain**		
No	80 (68%)	39 (33%)
Yes	18 (15%)	61 (52%)
Not within 6 months	20 (17%)	18 (15%)
**History with Back/Shoulder pain**		
No	33 (28%)	14 (12%)
Yes	61 (52%)	90 (76%)
Not within 6 months	24 (20%)	14 (12%)
**History with Joint/Limb pain**		
No	60 (51%)	13 (11%)
Yes	30 (25%)	100 (85%)
Not within 6 months	28 (24%)	5 (4%)
**History with Menstrual pain**		
No	67 (57%)	64 (54%)
Yes	17 (14%)	33 (28%)
Not within 6 months	34 (29%)	21 (18%)

Suppl. Table 1. Pain demographics in the control and pain patient groups.

Gender	Controls	Patients
Male	15 (13%)	15 (13%)
Female	103 (87%)	103 (87%)
**Education**		
Primary school	5 (4%)	21 (18%)
career college	21 (18%)	47 (40%)
Univ. of applied sciences	28 (24%)	31 (26%)
University	64 (54%)	19 (16%)
**Age**		
min	18 years	18 years
max	70 years	70 years
mean	43.9 years	44,3 years
**Work**		
Physical % of work		
Mean	22.97	37.20
Std.dev	25.73	28.46
Sitting % of working time		
Mean	60.93	47,54
Std. dev	31.57	31.32

Suppl. Table 2. Demographics of the patients and controls.

Use of painkillers	Controls	Patients
**Overcounter**		
Daily	1 (0.8%)	19 (16%)
Weekly	13 (11%)	31 (26%)
Monthly	43 (36%)	22 (19%)
Less frequently	58 (49%)	36 (31%)
Never	3 (2.5%)	10 (9%)
**Prescription**		
Daily	0	90 (76%)
Weekly	7 (6%)	14 (12%)
Monthly	9 (8%)	4 (3%)
Less frequently	53 (45%)	8 (7%)
Never	49 (42%)	2 (2%)
**Other CNS medications**		
Daily	10 (9%)	67 (57%)
Weekly	1 (0.8%)	3 (2.5%)
Monthly	2 (1.7%)	1 (0,8%)
Less frequently	6 (5%)	17 (14%)
Never	99 (84%)	30 (25%)

Suppl. Table 3. The use of analgesics in the control and patient groups. The figure shows the use of over-the-counter, prescription and other medications having effect on the central nervous system (CNS).

Supplemental Additional Analyses

These additional analyses were performed based on a request by one of the reviewers. In these analyses, we restricted the matched controls to those who had responded 0, 1 or 2 on BPI average or BPI now, and had reported current pain at 0, 1, or 2 on the additional pain intensity questions outside of BPI. 106 of the original 118 matched controls met these criteria. Next, we re-run the primary analyses using this more clearly pain-free group of controls and the whole original set of pain patients.

Analysis 3.1

The pain patients coloured a larger area of the body for both current pain (Mdn = 0.10 of the total body) and chronic pain (Mdn = 0.14 of the total body) conditions than the controls (Mdn = 0 and Mdn = 0.03, respectively). A Mann-Whitney test indicated this difference was statistically significant for both current ($r = 0.775$, $p < 0.001$) and chronic ($r = 0.607$, $p < 0.001$, p-values Holm-Bonferroni corrected) pain. These results agree with those produced with the original control sample with only minor differences in effect sizes. Thus, the conclusions are the same as after the original analysis.

Analysis 3.2.1:

Overall, the pain patients coloured more areas as sensitive for nociception ($M = 0.13$, $SD = 0.12$) than the current 106 controls ($M = 0.06$, $SD = 0.07$) ($U = 8588$, $p < 0.001$, $r = -0.33$). There was no

significant difference in the whole body colouring of tactile sensitivity (U = 6140, p = 0.9, r= -0.008) or hedonic sensitivity (U = 5140, p = 0.09, r= 0.13, p-values Holm-Bonferroni corrected for multiple comparisons). In two-way between-within ANOVAs on trimmed means for each separate ROI, there were no significant main effects of group (patient or control, ps >= 0.07). The main effect of condition (tactile, hedonic or nociceptive sensitivity) was significant for all ROIs (ps <= 0.03) apart from legs (p = 0.37) and feet (p = 0.17). The only significant interaction was for the lower torso (p=0.01). In pair-wise Mann-Whitney U-tests between the groups for each ROI and condition, the only significant differences were found in the reporting of nociceptive sensitivity in shoulders (U= 8200, p < 0.0001), arms (U= 8513, p < 0.0001), hands (U = 8192, p < 0.0001), and legs (U= 7622, p = 0.006). In all of these cases, the patients had coloured in more areas as being sensitive to nociception than controls. These results and the conclusions fully agree with those of the original analysis using the larger set of control subjects.

Analysis 3.3.

A two way between-within subjects ANOVA on trimmed means was conducted with a between-subject factor as group membership (2 levels: patient and control) and within-subject factor as emotion (7 levels). There was a significant main effect for group (F(1, 356.73)= 30.33 and p < 0.0001), such that on average the bodily area coloured by the pain patients (mean proportion coloured = 0.2, sd=0.23) was smaller than the area coloured by the controls (mean proportion coloured = 0.26, sd=0.24). There was also a significant main effect for emotion (F(6, 202.02)= 66.16 and p < 0.0001). There was no significant interaction between group and emotion (F(6, 202.78)=1.40 and p = 0.22). The least coloured body area was in the neutral emotional state (mean 0.087, SD 0.173), followed by surprise (mean 0.132, SD 0.172) and disgust (mean 0.187, SD 0.189).

Similar main effects of group and emotion were also found when analysing activations (F(1, 249.87) = 12.77, p = 0.001; F(6, 183.06) = 136.55, p < 0.0001) or deactivations (F(1, 147.52) = 31.31, p < 0.0001; F(6, 179.04) = 26.70, p < 0.0001) separately. The interaction between group and emotion was significant for deactivations (F(6, 179.04) = 6.15, p < 0.0001), but not for activations (F(6, 182.08)= 1.99, p = 0.13, all p-values Holm-Bonferroni corrected). While the exact test statistic values are slightly different from those in the primary analysis, no conclusions change when the more restrictive control population is used.

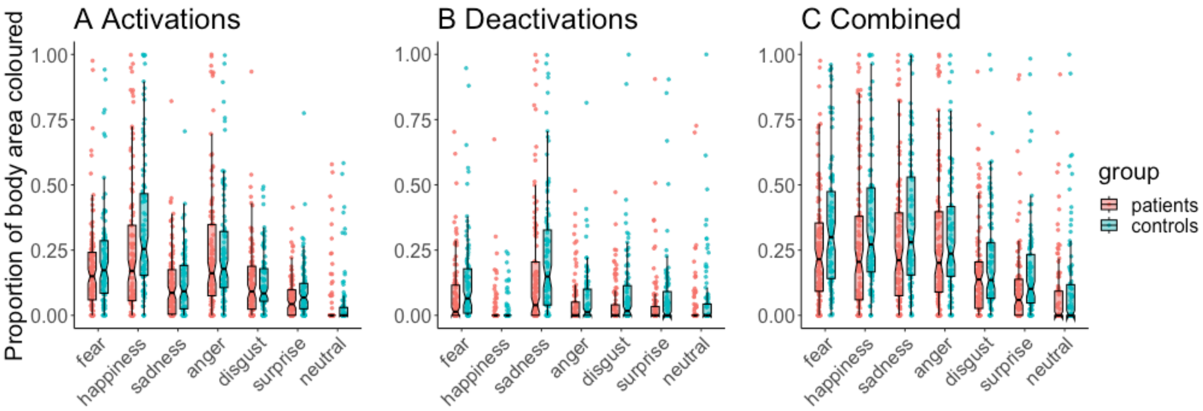

Figure 7 redrawn to display the comparison of the whole pain patient population (n=118) with the more strictly pain-free control population (n=106)

Analysis 3.3.1

To test whether the aggregate effect was localized on a specific body area, we analysed the total number of coloured (activation or deactivation) pixels in eight anatomically defined regions of interest (ROI). In the ROI analysis (Suppl. Figure 2), we ran a two-way between-within ANOVA on trimmed means for each body part, separately. There was a significant main effect of group for each ROI (all p-values < 0.02, Holm-Bonferroni-corrected) with stronger bodily feelings in the controls versus patients. For all ROIs, there was also a significant main effect of emotion (all p-values < 0.001, Holm-Bonferroni corrected). In contrast with the analysis run with the previous full set of controls, three body areas show weak but nonetheless statistically significant interaction between emotion and group membership: legs ($F(6, 176.04) = 3.45$, $p= 0.02$), feet ($F(6, 176.03) = 3.20$, $p = 0.037$) and hands ($F(6, 176.04) = 3.02$, $p = 0.047$

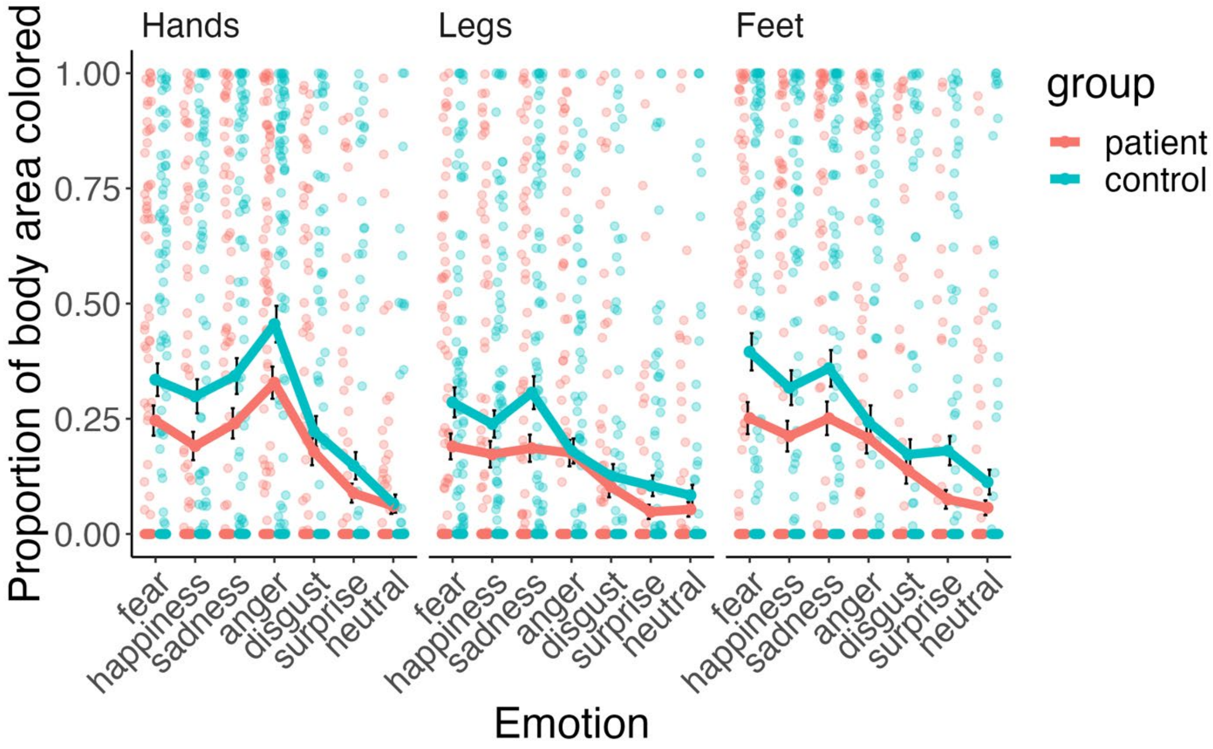

Extra supplemental figure depicting the three body areas with weak but significant interaction between group membership (pain patient or pain-free control) and emotion type.

Sensitivity Ratio

We were requested to replicate a “sensation ratio” score in the spirit of Hanley & Garland. We decided to calculate this based on hedonic and nociceptive sensitivity maps as 1) we did not have subjects reporting positive sensations they were currently experiencing in their body, 2) using pain maps would have directly biased the data towards the pain patients as they were selected to participate because they had intense pain maps. We re-conceptualized ”sensation ratio” as a “sensitivity ratio” and calculated it as

$$\frac{hedonic\ sensitivity\ colored\ pixels}{nociceptive + hedonic\ sensitivity\ colored\ pixels}.$$

Thus, values over 0.5 mean that the subject colored in more areas as hedonic sensitive and values under 0.5 mean that they colored in nociceptive sensitive areas.

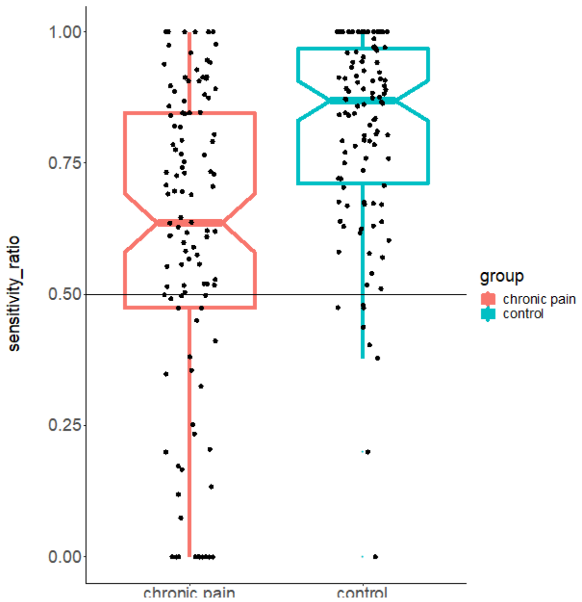

Both pain patients and controls reported more hedonic sensitive than nociceptive sensitive areas (sensitivity ratio > 0.5) on average. Controls reported on average significantly higher sensitivity ratios than pain patients.

mean pain = 0.5961364, mean control = 0.8143968, Welch two-sample t-test: t(182.26)=-6.4494, p = 9.795e-10

Clear group difference, as can be seen in the figure, individual level responses are not as neatly differentiated.
